# The complete chloroplast genome sequence of *Kadsura ananosma*

**DOI:** 10.1080/23802359.2020.1715866

**Published:** 2020-01-22

**Authors:** Liangyan Liu, Yupin Fu, Yunqing Li, Yi Wang

**Affiliations:** a College of Agronomy and Biotechnology, Yunnan Agriculture University, Kunming, Yunnan, People’s Republic of China;; b Laboratory of Forest Plant Cultivation and Utilization, Yunnan Academy of Forestry, Kunming, Yunnan, People’s Republic of China

**Keywords:** *Kadsura ananosma*, chloroplast, Illumina sequencing, phylogenetic analysis

## Abstract

The first complete chloroplast genome (cpDNA) sequence of *Kadsura ananosma* was determined from Illumina HiSeq pair-end sequencing data in this study. The cpDNA is 145,903 bp in length, contains a large single-copy region (LSC) of 94,757 bp and a small single-copy region (SSC) of 18,042 bp, which were separated by a pair of inverted repeats (IR) regions of 16,552 bp. The genome contains 125 genes, including 82 protein-coding genes, 8 ribosomal RNA genes, and 35 transfer RNA genes. Further phylogenomic analysis showed that *K. ananosma* and *Kadsura coccinea* clustered in a clade in Schisandraceae family.

*Kadsura ananosma* is the species with the family Schisandraceae, native to Xishuangbanna of Yunnan, China (Yang, Pu, et al. 2010). The fruits can be eaten as wild fruit (Chen et al. 2001). Its roots, vines and fruits are precious Dai medicines, which have the functions of astringent, promoting body fluid, tonifying kidney and tranquilizing heart (Chen et al. 2016). Lignans and triterpenoids isolated from *K. ananosma* have antioxidant, antitumor, and anti-HIV activities (Yang, Wen, et al. 2010). Therefore, *K. ananosma* has huge application prospects. However, there has been no genomic studies on *K. ananosma.*

Herein, we reported and characterized the complete *K. ananosma* plastid genome. The GenBank accession number is MN823697. One *K. ananosma* individual (specimen number: 201905067) was collected from Kunming arboretum, Yunnan Academy of Forestry, Yunnan Province of China (25°14′21ʺN, 102°75′19ʺE). The specimen is stored at Yunnan Academy of Forestry Herbarium, Kunming, China, and the accession number is YAFH0012985. DNA was extracted from its fresh leaves using DNA Plantzol Reagent (Invitrogen, Carlsbad, CA, USA).

Paired-end reads were sequenced by using Illumina HiSeq system (Illumina, San Diego, CA). In total, about 23.9 million high-quality clean reads were generated with adaptors trimmed. Aligning, assembly, and annotation were conducted by CLC *de novo* assembler (CLC Bio, Aarhus, Denmark), BLAST, GeSeq (Tillich et al. 2017), and GENEIOUS v 11.0.5 (Biomatters Ltd, Auckland, New Zealand). To confirm the phylogenetic position of *K. ananosma*, other six species of *Schisandraceae* family from NCBI were aligned using MAFFT v.7 (Katoh and Standley 2013). The auto algorithm in the MAFFT alignment software was used to align the nine complete genome sequences and the G-INS-i algorithm was used to align the partial complex sequences. The maximum likelihood (ML) bootstrap analysis was conducted using RAxML (Stamatakis 2006); bootstrap probability values were calculated from 1000 replicates. *Nuphar advena* (DQ354691) and *Nuphar longifolia* (MH050795) were served as the out-group.

The complete *K. ananosma* plastid genome is a circular DNA molecule with the length of 145,903 bp, contains a large single-copy region (LSC) of 94,757 bp and a small single-copy region (SSC) of 18,042 bp, which were separated by a pair of inverted repeats (IR) regions of 16,552 bp. The overall GC content of the whole genome is 39.7%, and the corresponding values of the LSC, SSC, and IR regions are 38.6%, 35.0%, and 45.5%, respectively. The plastid genome contained 125 genes, including 82 protein-coding genes, 8 ribosomal RNA genes, and 35 transfer RNA genes. Phylogenetic analysis showed that *K. ananosma* and *K. coccinea* clustered in a unique clade in *Schisandraceae* family (Figure 1). The determination of the complete plastid genome sequences provided new molecular data to illuminate the *Schisandraceae* family evolution.

**Figure 1.**
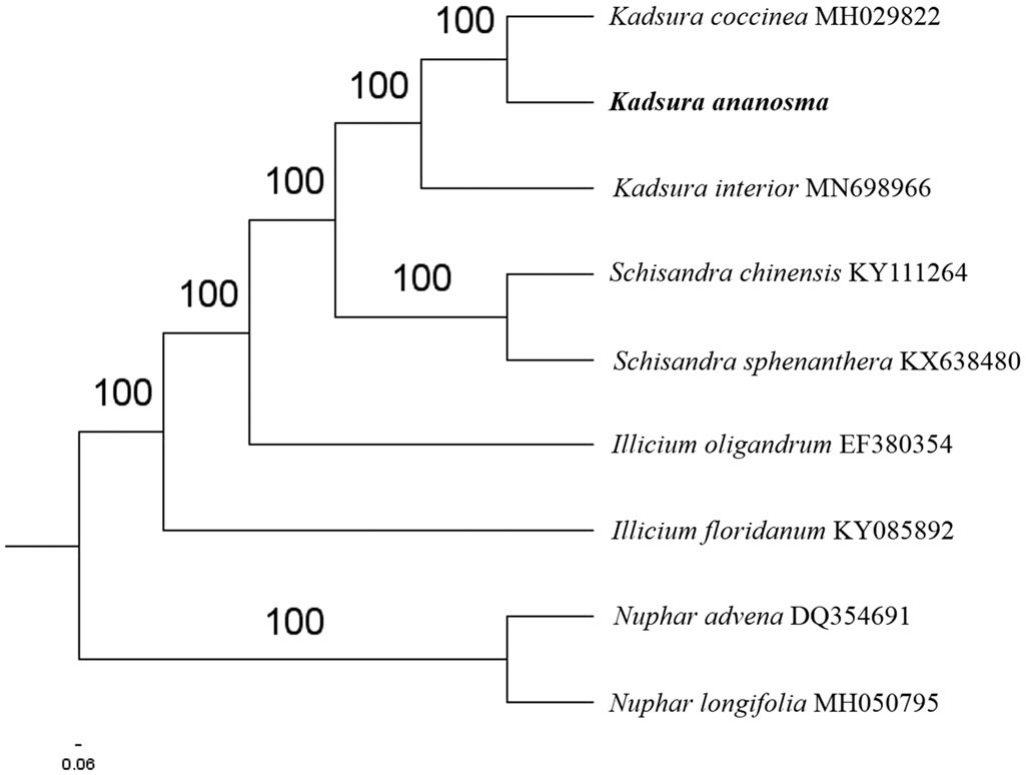
The maximum-likelihood tree based on the seven chloroplast genomes of *Schisandraceae* genus. The bootstrap value based on 1000 replicates is shown on each node.
